# Supplementary figures and images for: Synthesis and Biological Evaluation of Novel Folic Acid Receptor-Targeted, β-Cyclodextrin-Based Drug Complexes for Cancer Treatment
Source: PLoS One. 2013 May 2;8(5):e62289. doi: 10.1371/journal.pone.0062289 (PMC3642146; doi:10.1371/journal.pone.0062289)

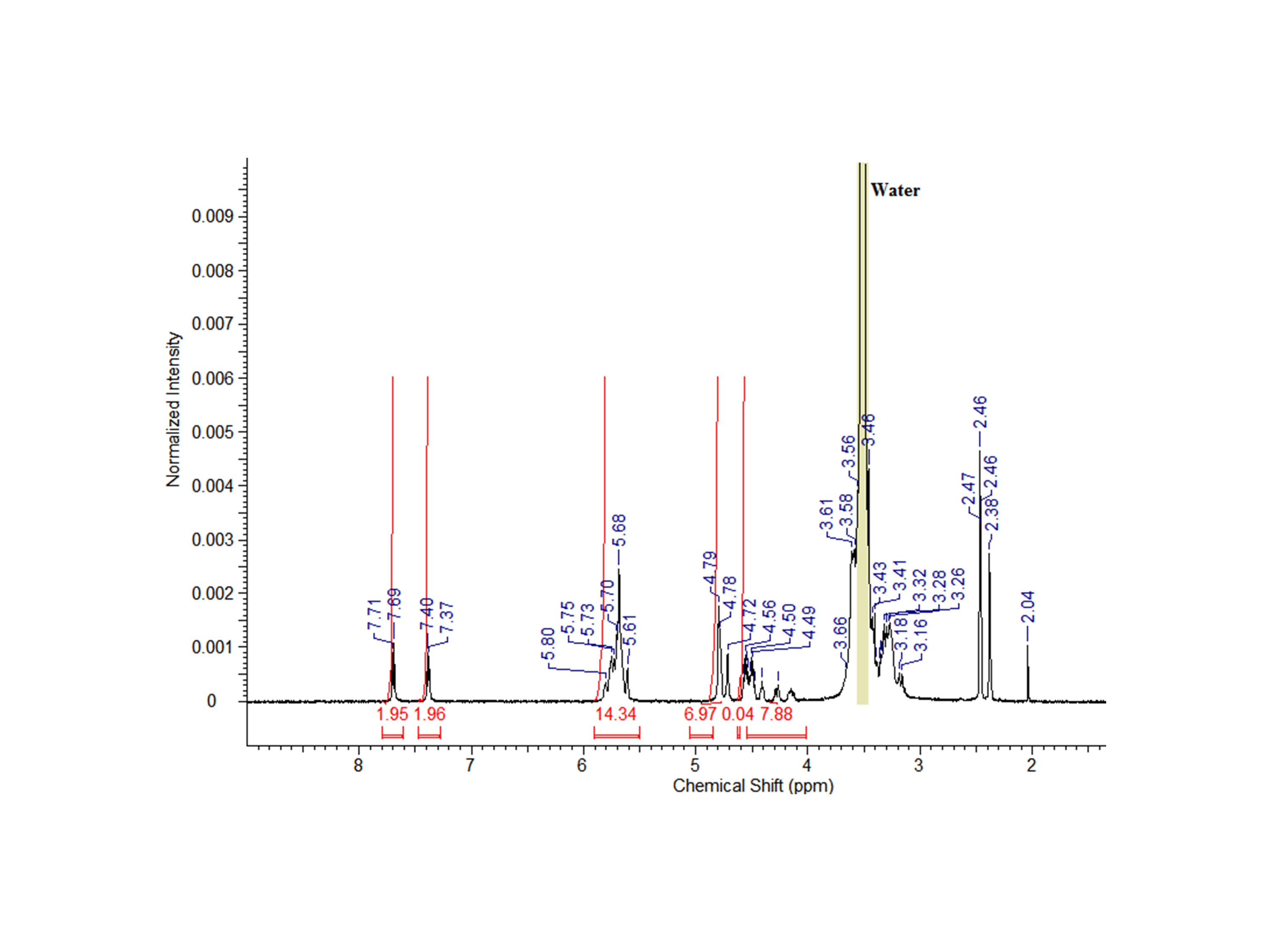

Supplement: Figure S1 — The 1H-NMR spectrum of Ts-CD (400 MHz, D2O). (TIF) [file pone.0062289.s001.tif]

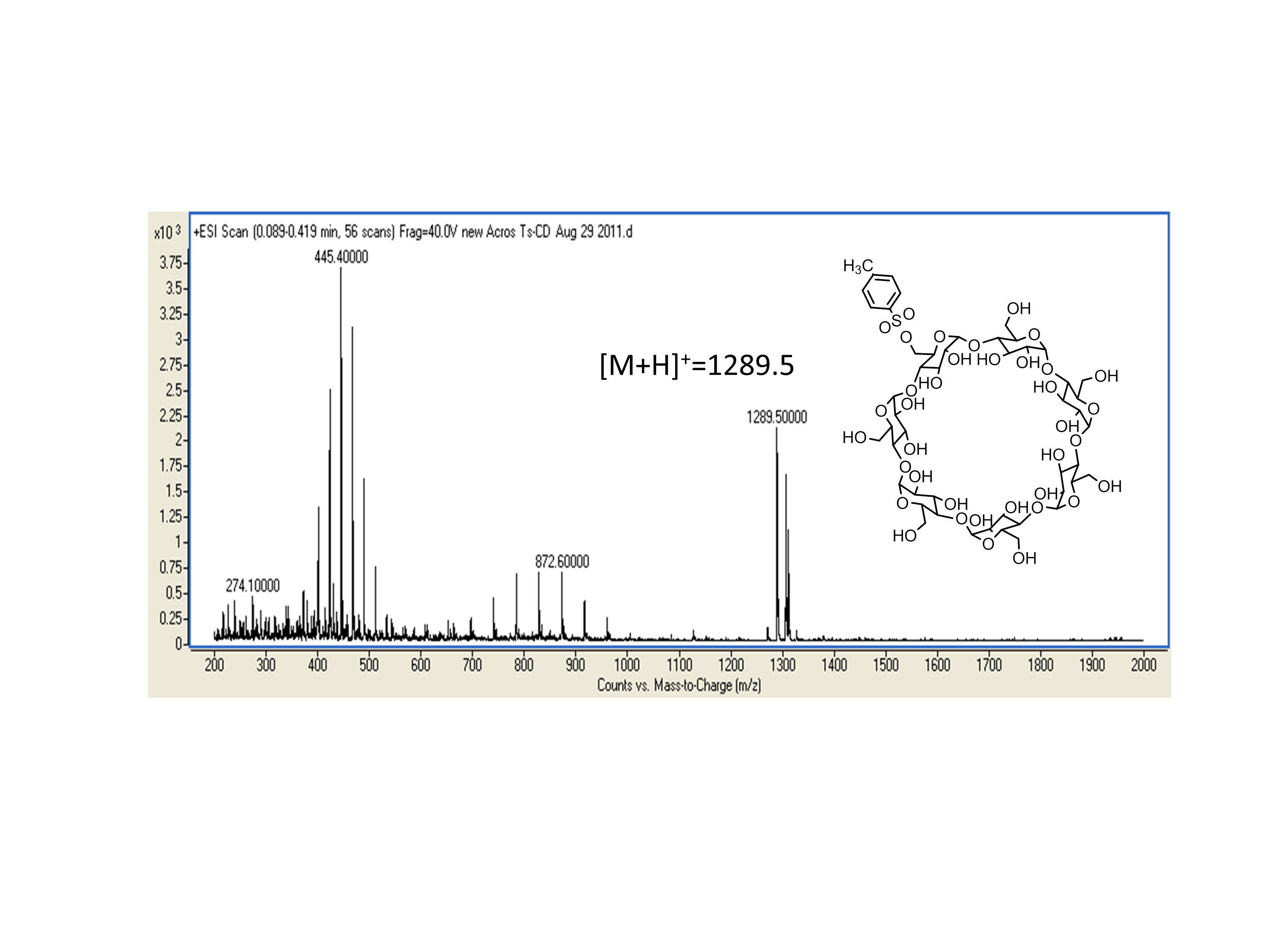

Supplement: Figure S2 — The ESI-MS spectrum of Ts-CD. (TIF) [file pone.0062289.s002.tif]

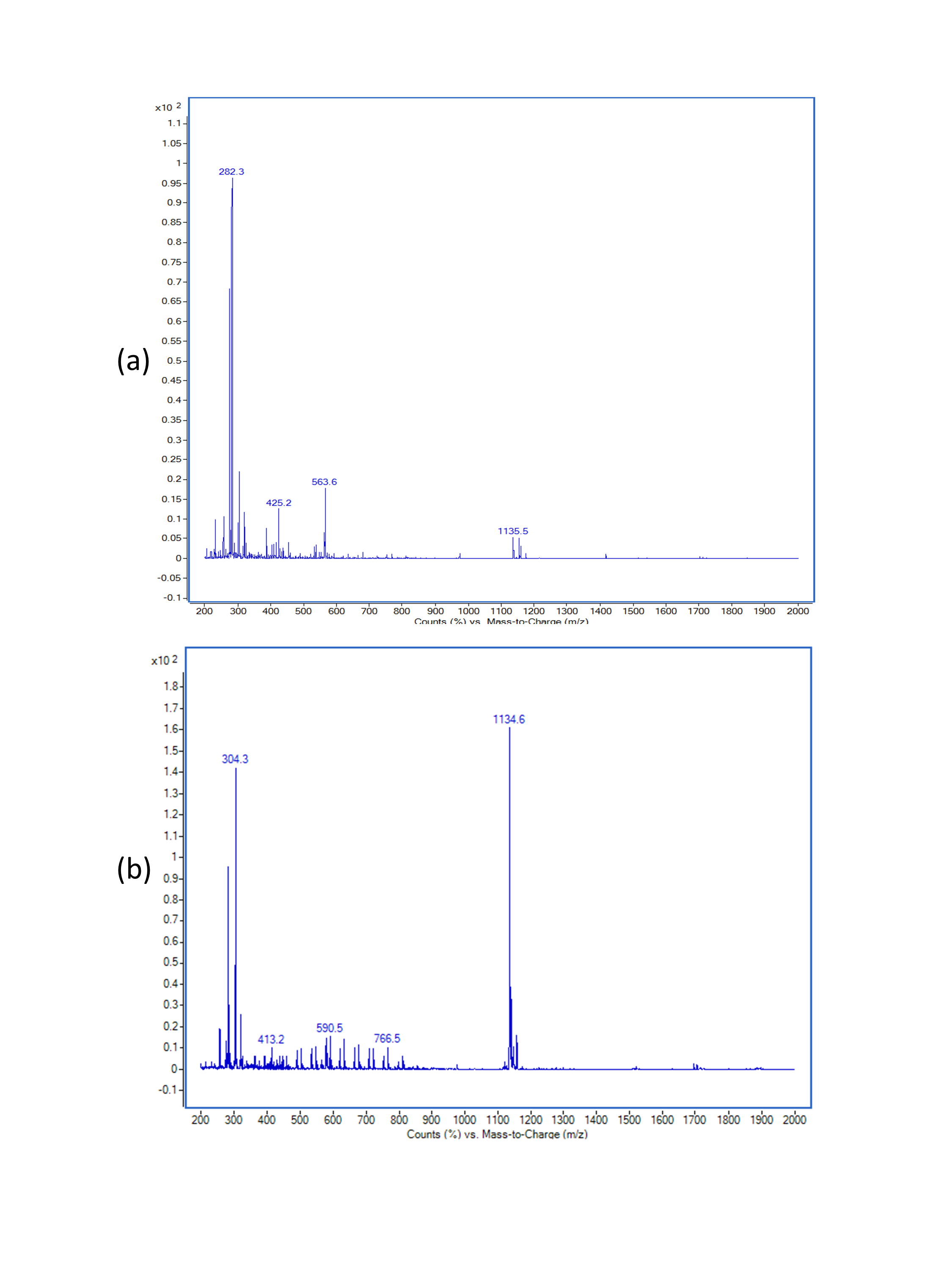

Supplement: Figure S3 — The ESI spectrum of β-CD (a) and NH2-CD (b). (TIF) [file pone.0062289.s003.tif]

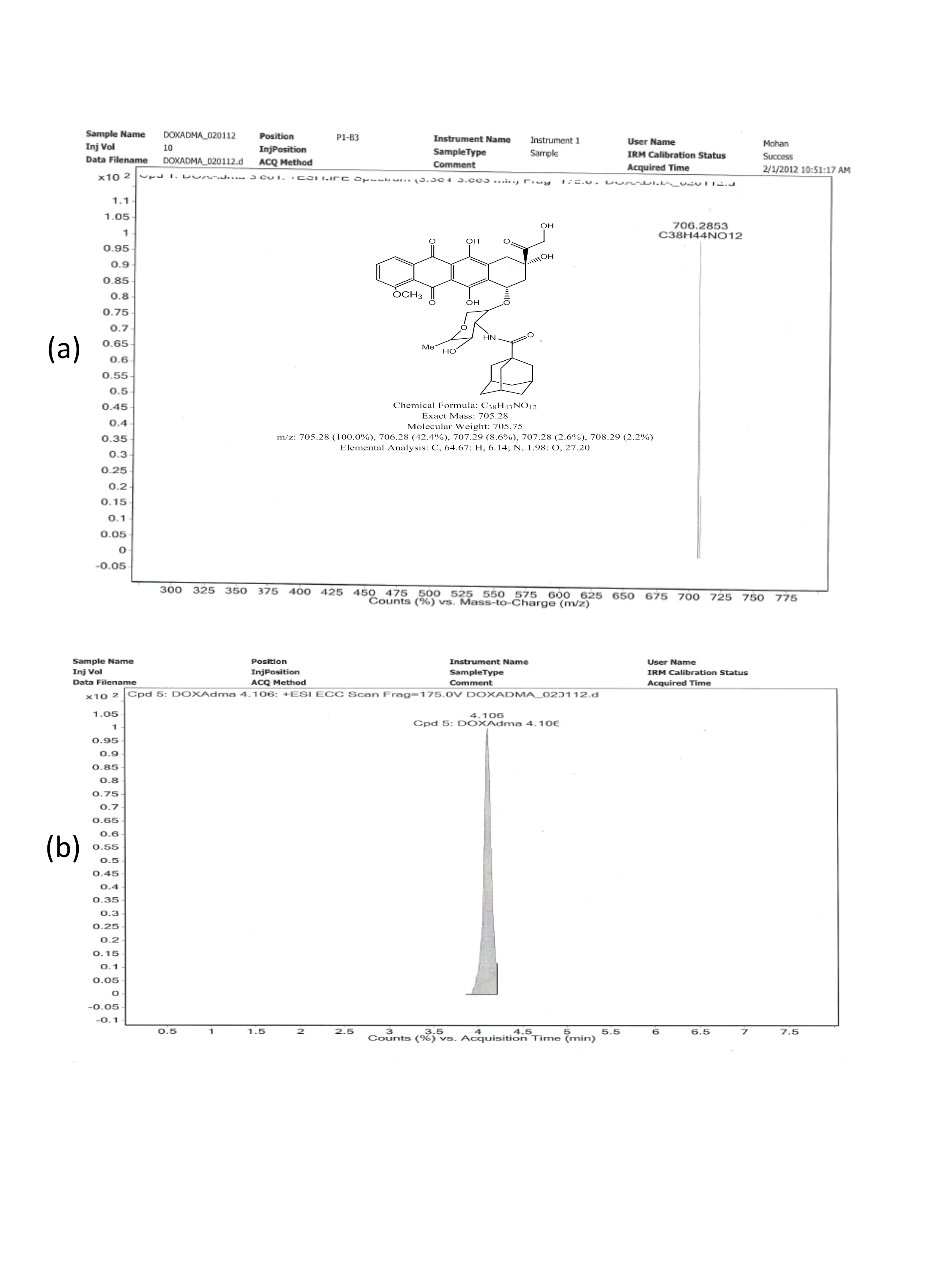

Supplement: Figure S4 — LC-MS spectra of Ada-DOX (a & b). (TIF) [file pone.0062289.s004.tif]

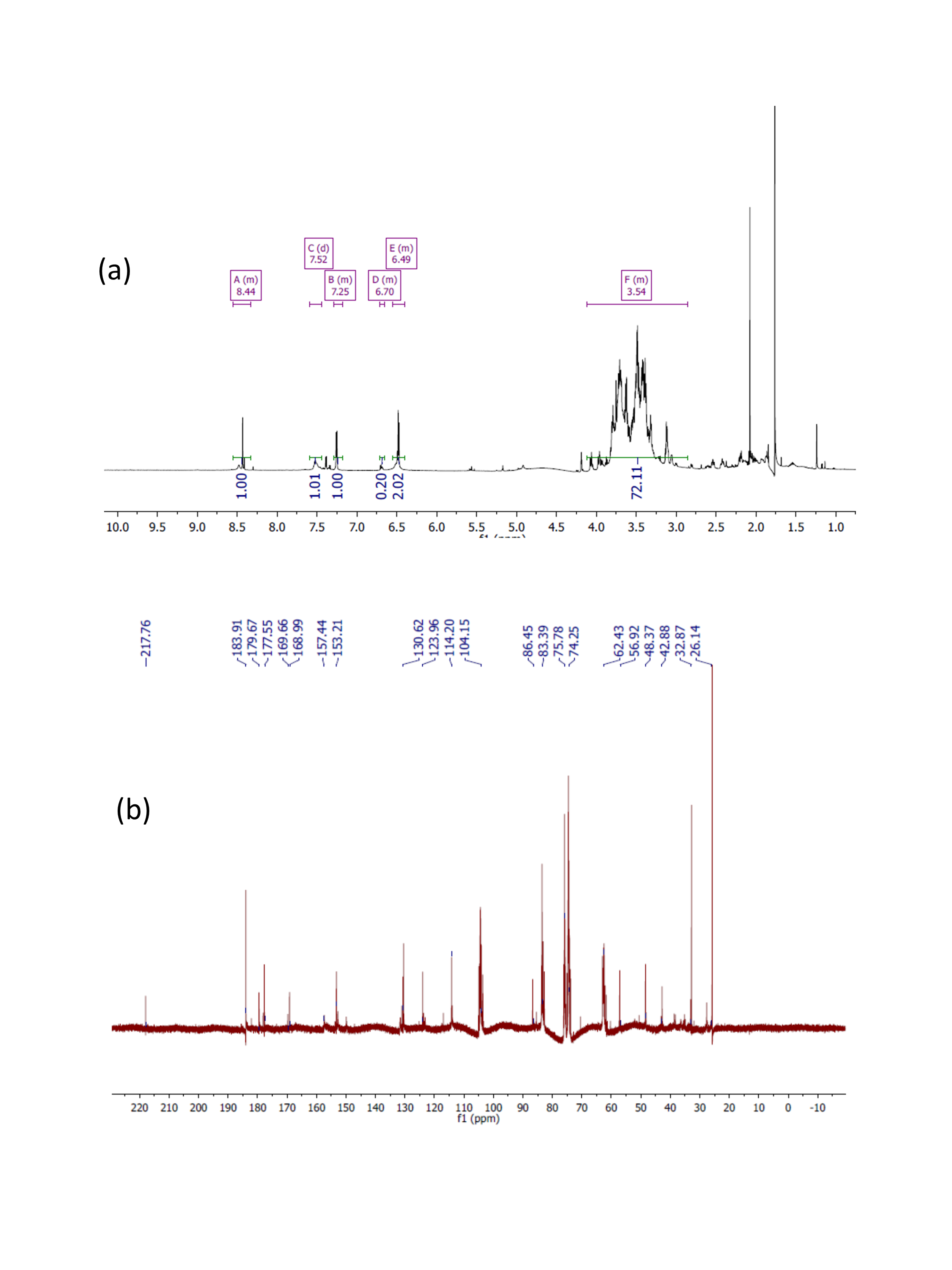

Supplement: Figure S5 — The 1H-NMR (a, 800 MHz, D2O) and 13C-NMR (b, 201 MHz, D2O) spectra of γ-FACD. (TIF) [file pone.0062289.s005.tif]

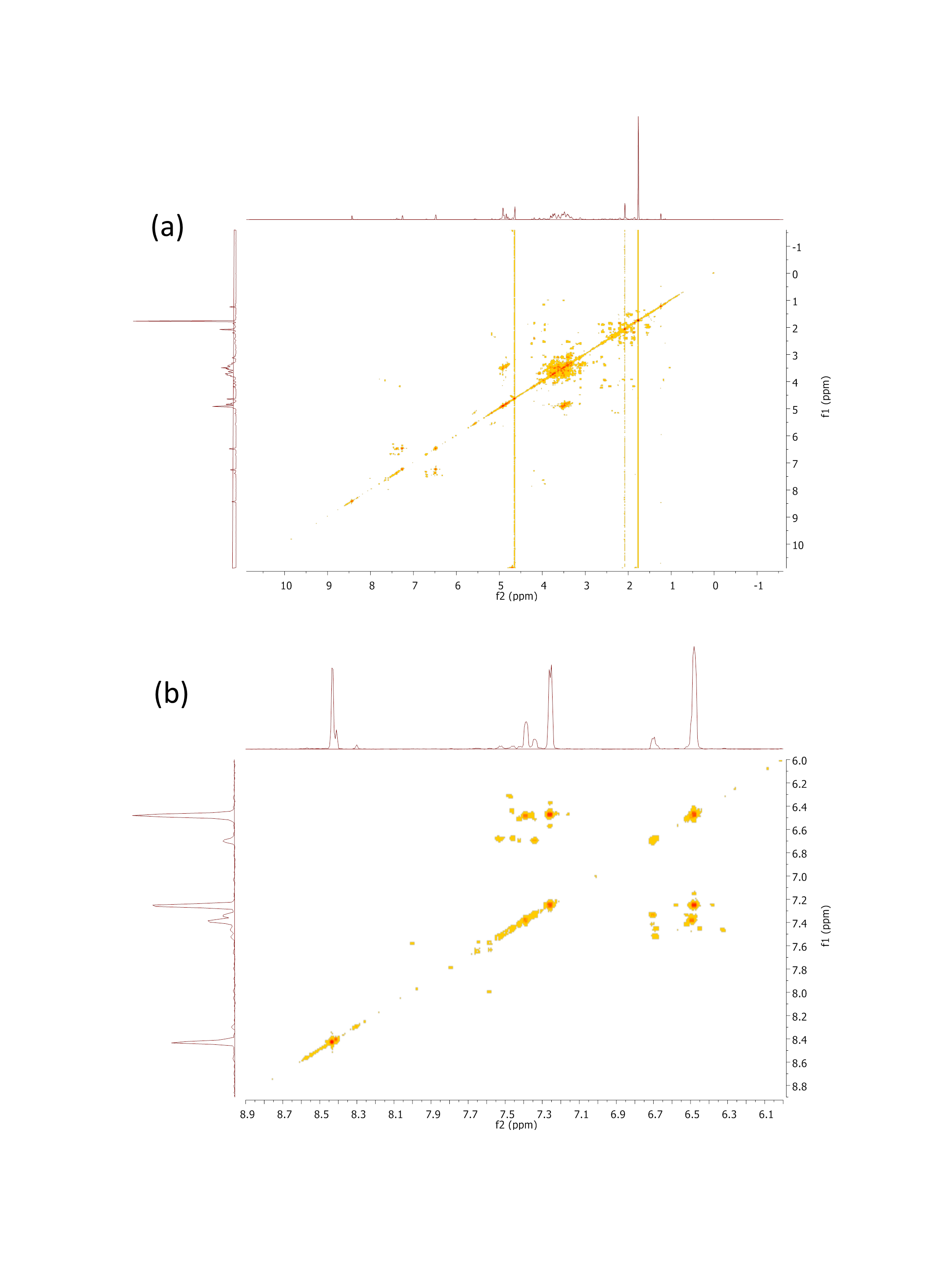

Supplement: Figure S6 — The g-COSY spectra of γ-FACD (a, 600 MHz, D2O, and when zoomed within 6–8 ppm, b). (TIF) [file pone.0062289.s006.tif]

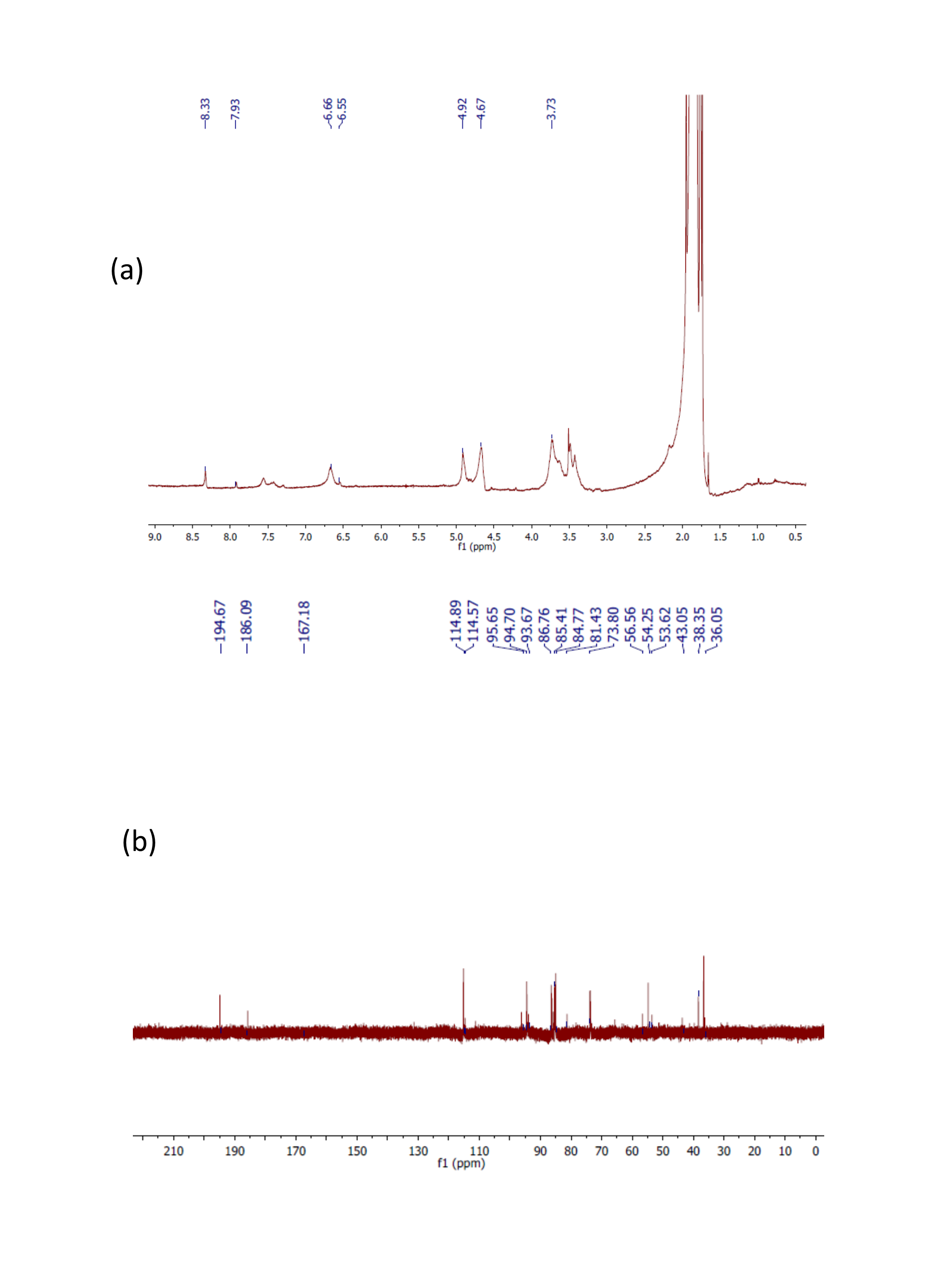

Supplement: Figure S7 — The 1H-NMR (a, 600 MHz, D2O) and 13C-NMR (b, 201 MHz, D2O) spectra of α-FACD. (TIF) [file pone.0062289.s007.tif]

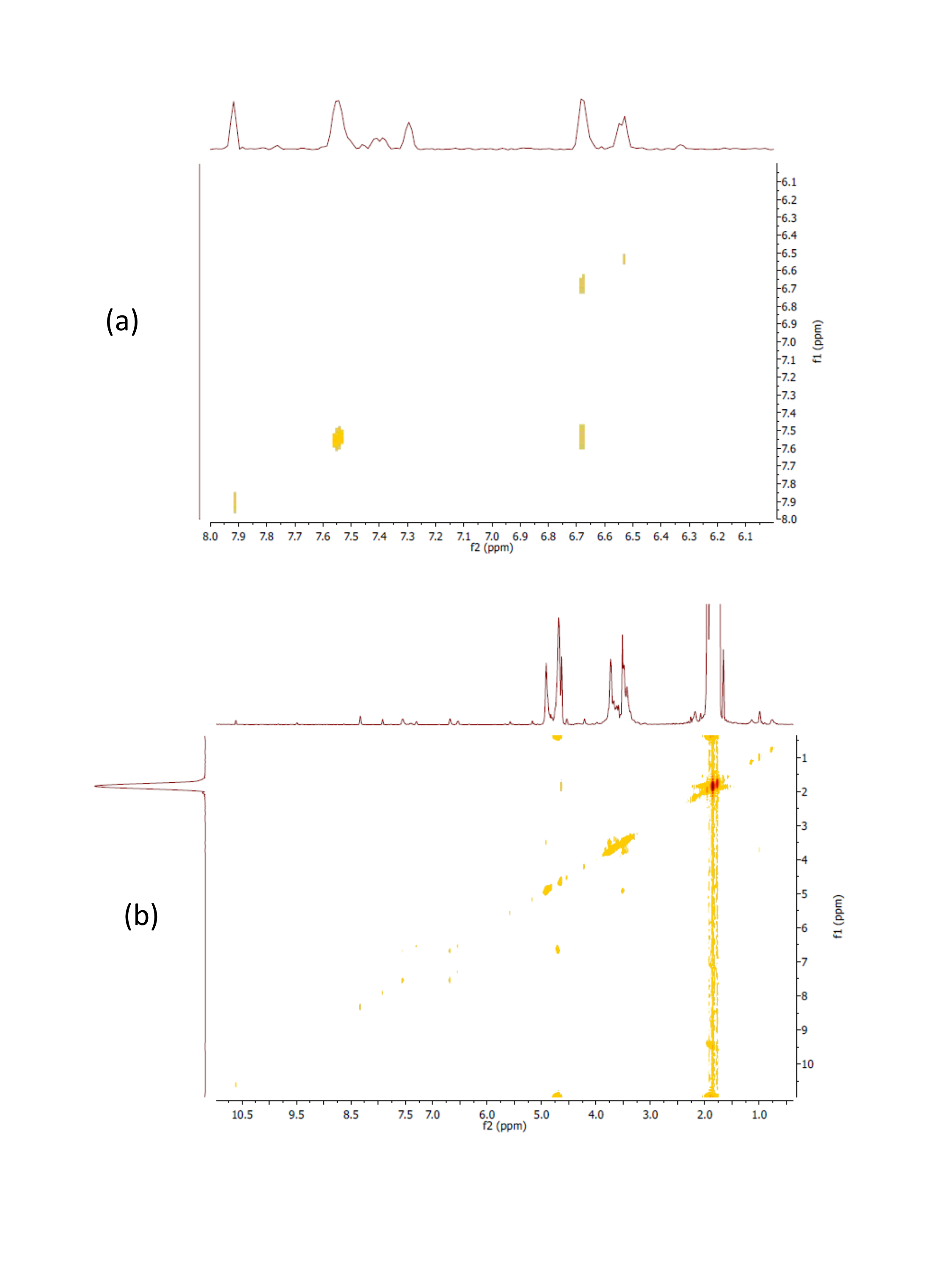

Supplement: Figure S8 — The g-COSY spectra of α-FACD (a, 600 MHz, D2O; when zoomed within 6–8 ppm, b). (TIF) [file pone.0062289.s008.tif]

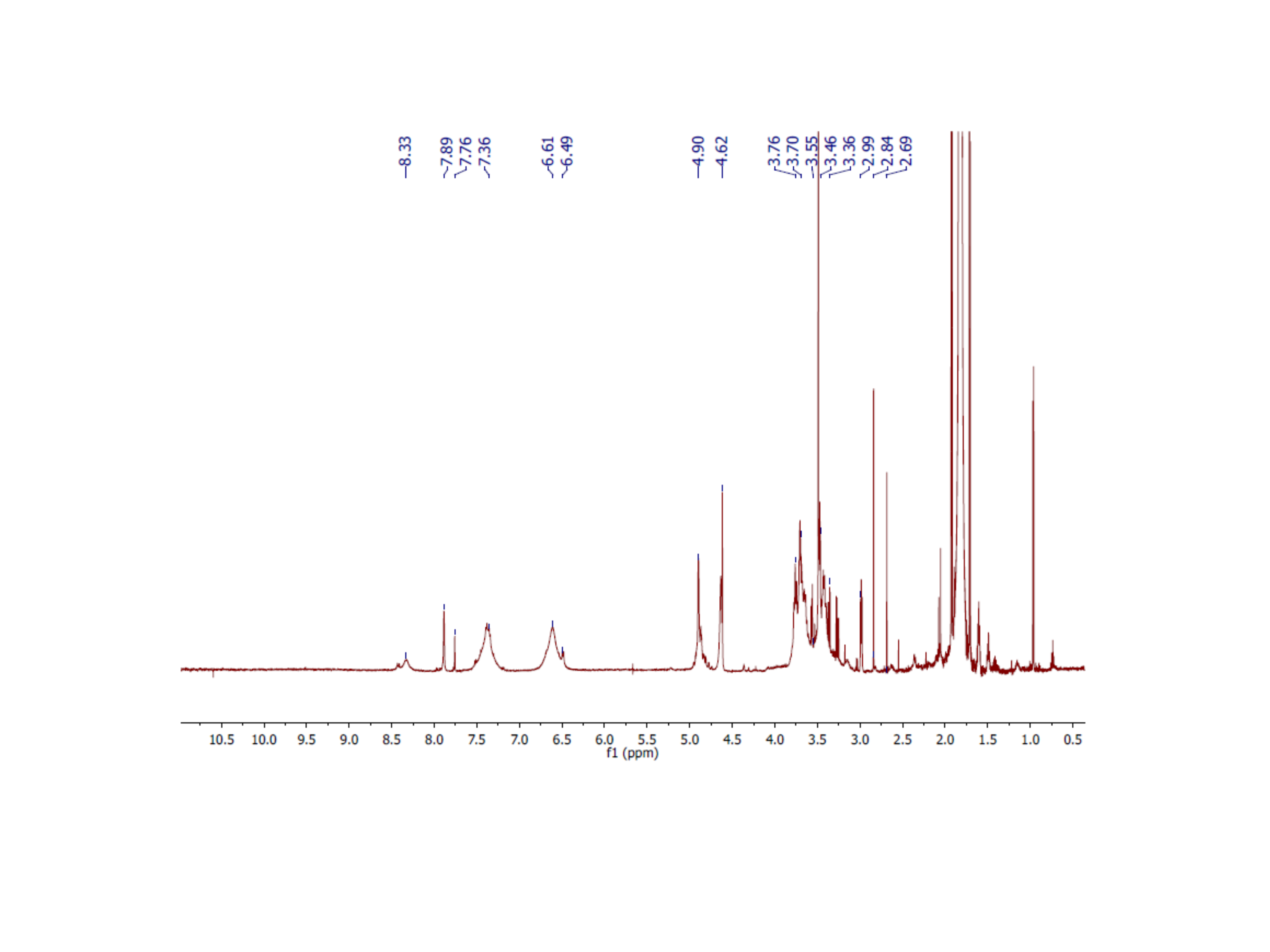

Supplement: Figure S9 — The 1H-NMR spectrum of FA-diCD (600 MHz, D2O). (TIF) [file pone.0062289.s009.tif]

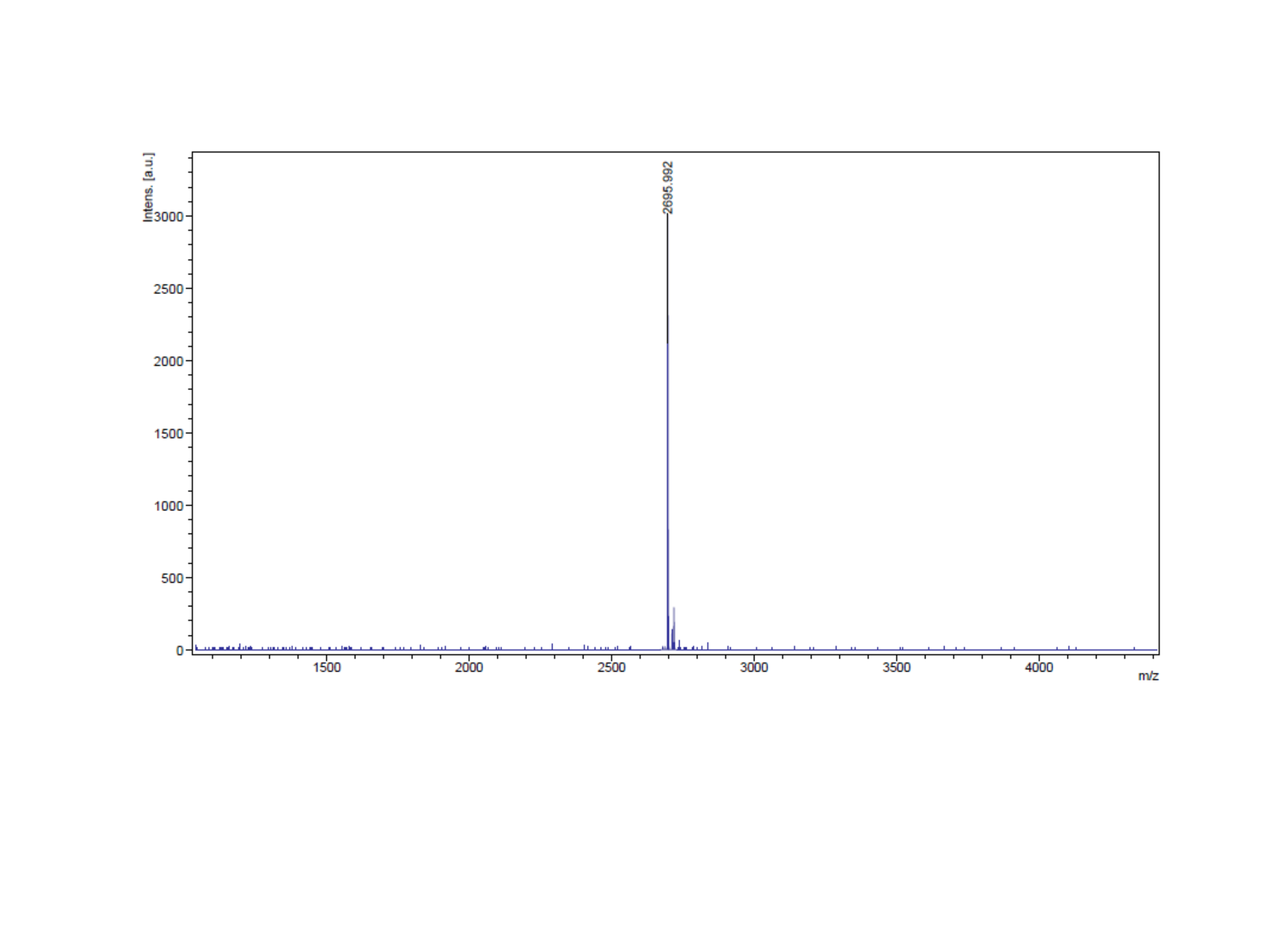

Supplement: Figure S10 — The original HR-MALDI-TOF spectrum of FA-diCD. (TIF) [file pone.0062289.s010.tif]

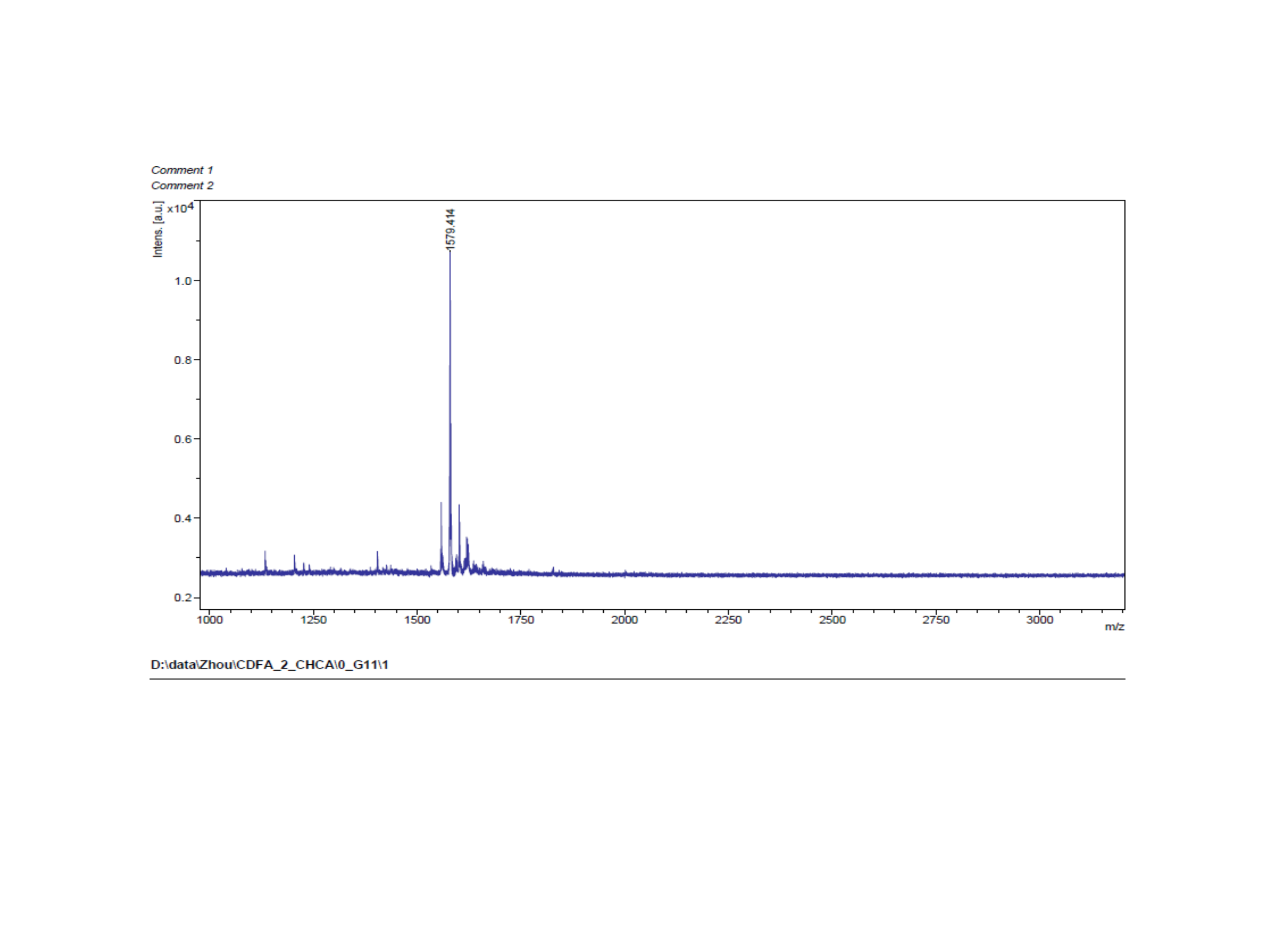

Supplement: Figure S11 — The original HR-MALDI-TOF spectrum of γ-FACD. (TIF) [file pone.0062289.s011.tif]

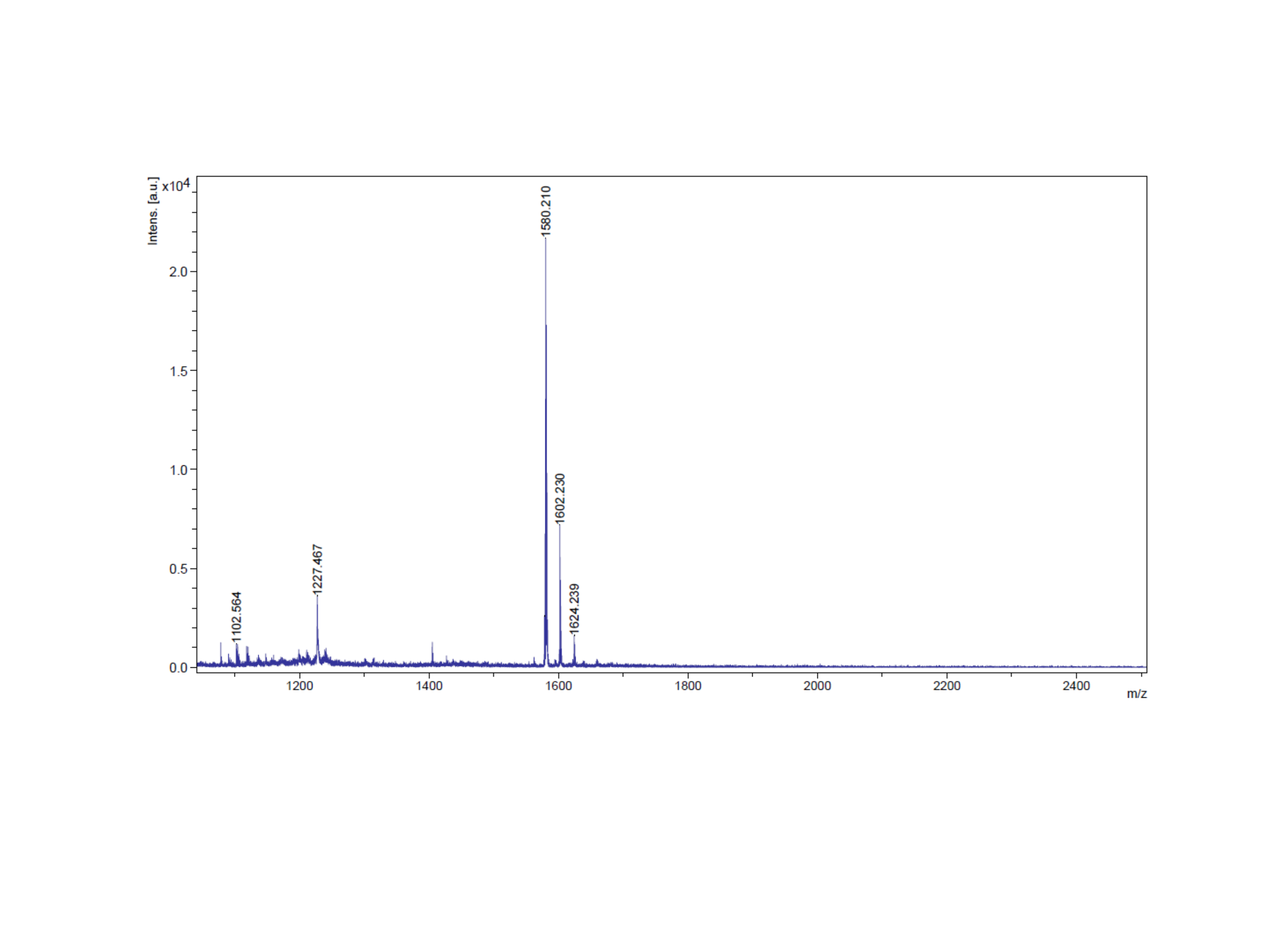

Supplement: Figure S12 — The original HR-MALDI-TOF spectrum of α-FACD. (TIF) [file pone.0062289.s012.tif]

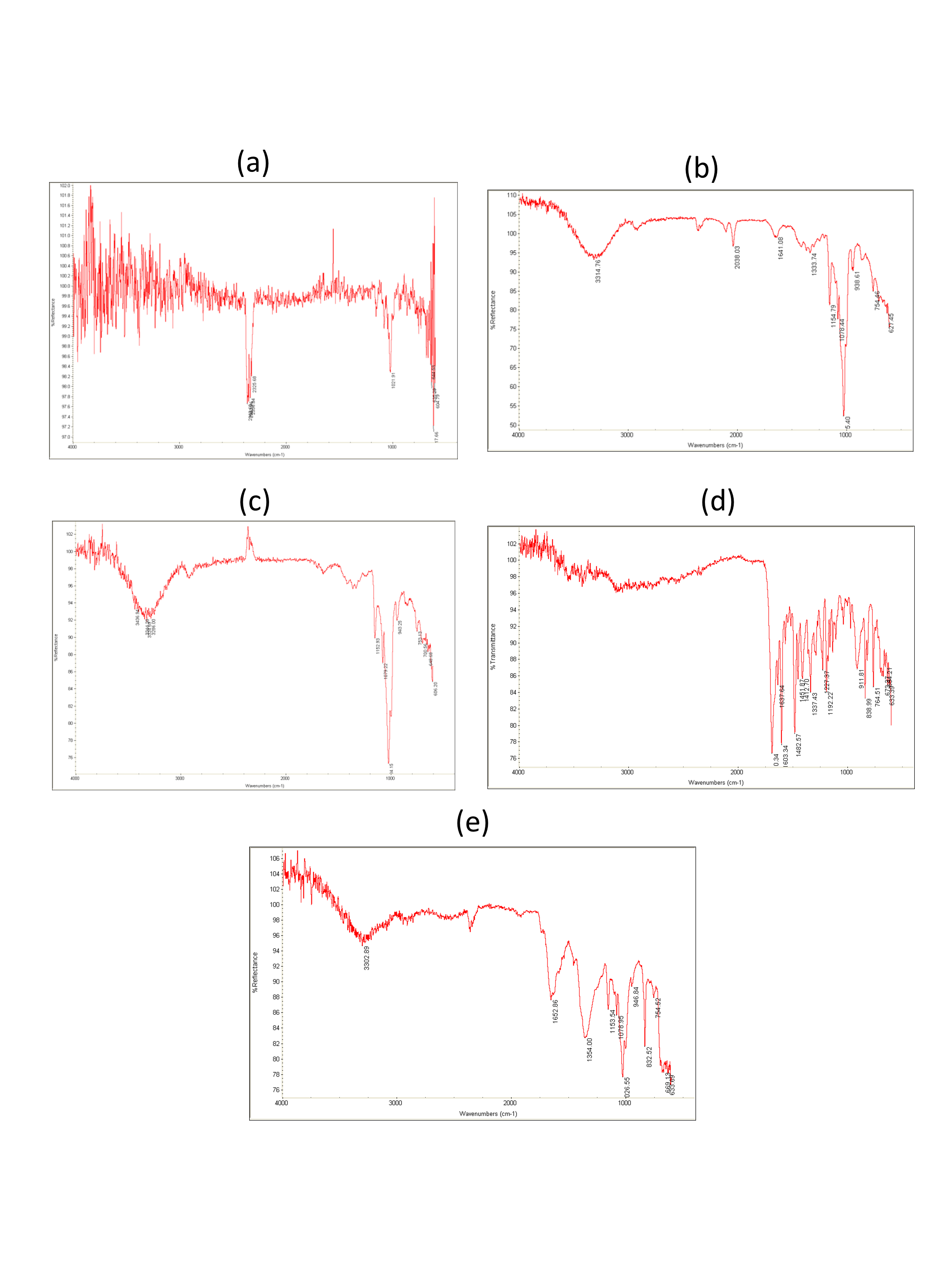

Supplement: Figure S13 — The FTIR spectra of β-CD (a), N3-CD (b), NH2-CD (c), FA (d), and γ-FACD (e). (TIF) [file pone.0062289.s013.tif]

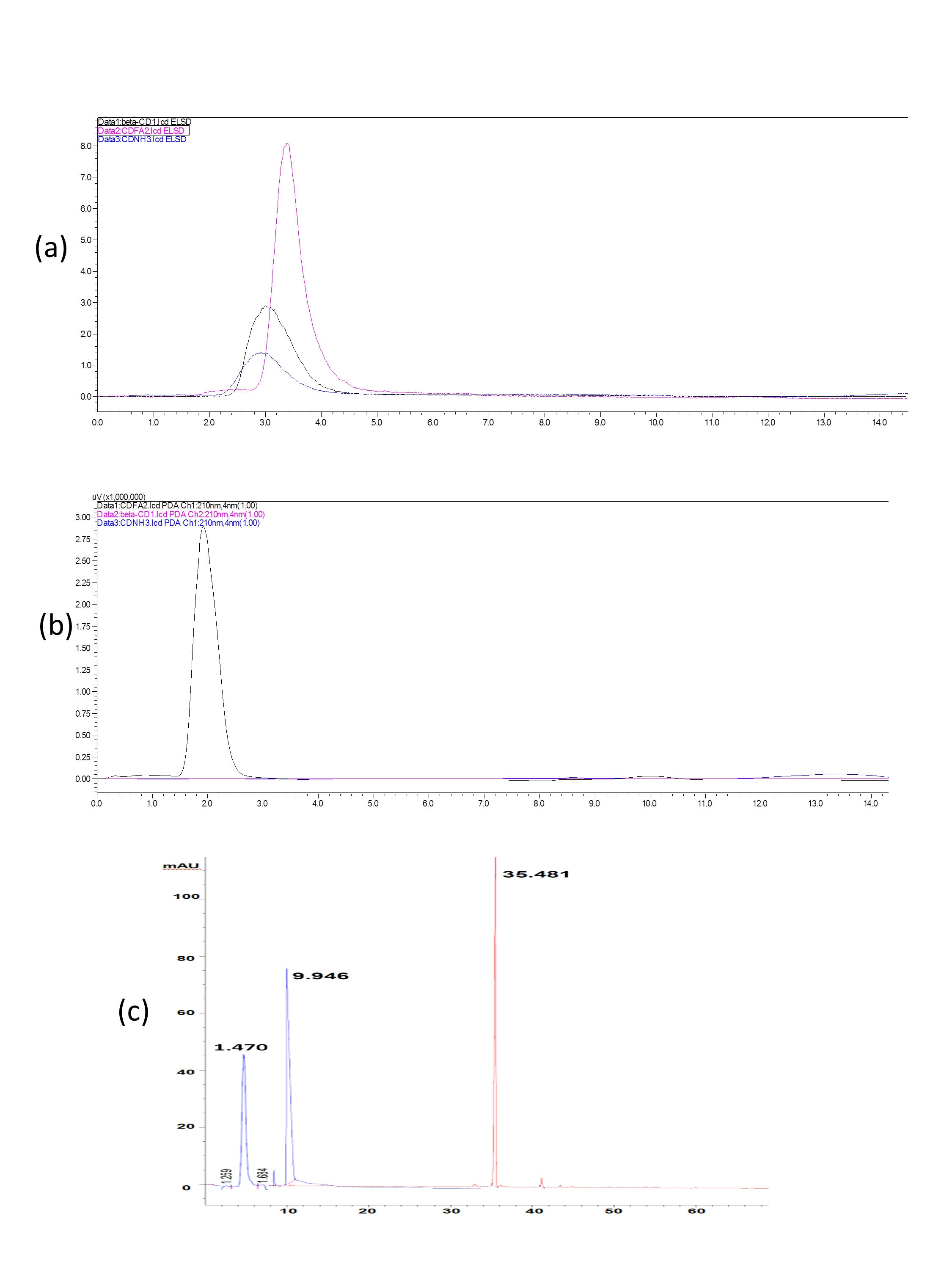

Supplement: Figure S14 — (a) The HPLC-ELSD chromatogram of β-CD (black), NH2-CD (blue) and γ-FACD (Magenta); (b) HPLC-UV chromatogram of β-CD (Magenta), NH2-CD (blue) and γ-FACD (black); and (c) HPLC-DAD chromatograms of Ada-Dox (35 min), Dox (9 min) and FACD-Ada-Dox (1.5 min). (TIF) [file pone.0062289.s014.tif]
